# Bezold–Jarisch reflex mediated syncope in pulmonary arterial hypertension: An illustrative case series

**DOI:** 10.1002/pul2.12147

**Published:** 2022-10-01

**Authors:** Kolade M. Agboola, Gurukripa N. Kowlgi, Ibolya Csecs, Hilary M. DuBrock, Hector R. Cajigas, Thomas G. Allison

**Affiliations:** ^1^ Department of Cardiovascular Medicine Mayo Clinic Rochester Minnesota USA; ^2^ Department of Medicine Jacobi Medical Center, Albert Einstein College of Medicine New York New York USA; ^3^ Department of Internal Medicine, Division of Pulmonary and Critical Care Medicine Mayo Clinic Rochester Minnesota USA; ^4^ Department of Pediatric and Adolescent Medicine, Division of Pediatric Cardiology Mayo Clinic Rochester Minnesota USA

**Keywords:** Bezold–Jarisch reflex, cardiopulmonary exercise testing, pulmonary arterial hypertension, syncope

## Abstract

We present a novel description of Bezold–Jarisch Reflex (BJR) during cardiopulmonary exercise testing (CPET) in three young female patients with Group 1 pulmonary arterial hypertension (PAH). These three cases presented within 26 months, representing only 0.8% of 11,387 tests on patients with PAH undergoing CPET during this time frame.

The Bezold–Jarisch reflex (BJR) is a cardioinhibitory phenomenon triggered by the activation of ventricular mechanoreceptors resulting in an intense parasympathetic response characterized by bradycardia, hypotension, atrioventricular (AV) block or asystole, and syncope. Depressor reflexes in the heart were first described by von Bezold in 1867 and later reinforced by Jarisch.^1^ The left ventricular inferoposterior wall contains sensory receptors with vagal afferent pathways that respond to both mechanical and chemical stimuli.^2^ As such, the range of potential triggers is quite broad, ranging from acute myocardial ischemia, coronary angiography, and the use of intra‐arterial nitroglycerin.^1^, ^2^ Exercise‐related BJR syncope has been described in a series of patients without structural heart disease, although this is uncommon.^3^ It likely plays a role in postexercise collapse in athletes.^4^ We propose the BJR as a potential mechanism of syncope during exercise in patients with pulmonary arterial hypertension (PAH) and describe illustrative cases.

In November 2018 we observed a dramatic case of syncope during cardiopulmonary exercise testing (CPET) in a young, female patient with Group 1 PAH. We reviewed our subsequent experience through January 2021 to determine the frequency of occurrence of syncope during CPET in patients with PAH and identified two additional cases meeting criteria for BJR syncope. The absence of AV nodal blocking agents at the time of CPET was confirmed for each patient, as well as the absence of prior tachy or brady arrhythmia history. We reviewed medical records and CPET results for the cases in detail to confirm the mechanism of syncope. Patients were referred for a clinically indicated CPET and consented to allow their data to be used for research purposes according to Minnesota Statute (§144.335). Query of the exercise testing database was conducted under IRB # 17‐004494. Chart review of the cases was performed electronically by primary and corresponding authors.

The index event involved a 21‐year‐old female patient with a known history of idiopathic Group 1 PAH. She was initially diagnosed at age 19 after presenting with recurrent syncope. At that time of the index CPET, she had been recently transitioned off treprostinil infusion onto dual‐oral therapy with tadalafil and ambrisentan. Transthoracic echocardiogram demonstrated mild right ventricular enlargement with mildly decreased systolic function, estimated right ventricular systolic pressure 50 mmHg, and averaged right ventricular free wall longitudinal strain −21%.

The patient underwent CPET using the standard Mayo treadmill protocol.^5^ Exercise capacity was limited with 7.2 min of exercise to partial completion of a peak workload of 3.0 MPH/12.5% grade (8.5 metabolic equivalents). Resting heart rate (HR) was 89 bpm, resting blood pressure (BP) 90/62 mmHg. Peak HR was 193 bpm, peak BP 142/60 mmHg, peak oxygen consumption (V̇O_2_) 19.2 ml/min/kg = 51% predicted,^6^ and V̇_E_/V̇CO_2_ slope 36.3. Exercise was terminated due to fatigue, as well as lightheadedness and dizziness. At 0:56 of active recovery at 1.7 MPH/0% grade, the patient was noted to have a 3‐beat run of nonsustained ventricular tachycardia (NSVT), immediately followed by a slow junctional rhythm with baseline right bundle branch morphology (Figure 1). The HR abruptly fell from 184 to 27 bpm within 5 beats. The patient experienced syncope and fortunately was caught by the exercise physiologist monitoring the study. While attending to the patient, BP could not be recorded until after the resolution of the event. NSVT was not thought to be the cause of syncope but rather an escape rhythm as the HR abruptly slowed. HR recovered spontaneously to 145 bpm by 1:48 postexercise, at which time BP had returned to 146/60 mmHg. This abrupt drop in HR, followed by syncope, was felt to be consistent with BJR syncope.

**Figure 1:**
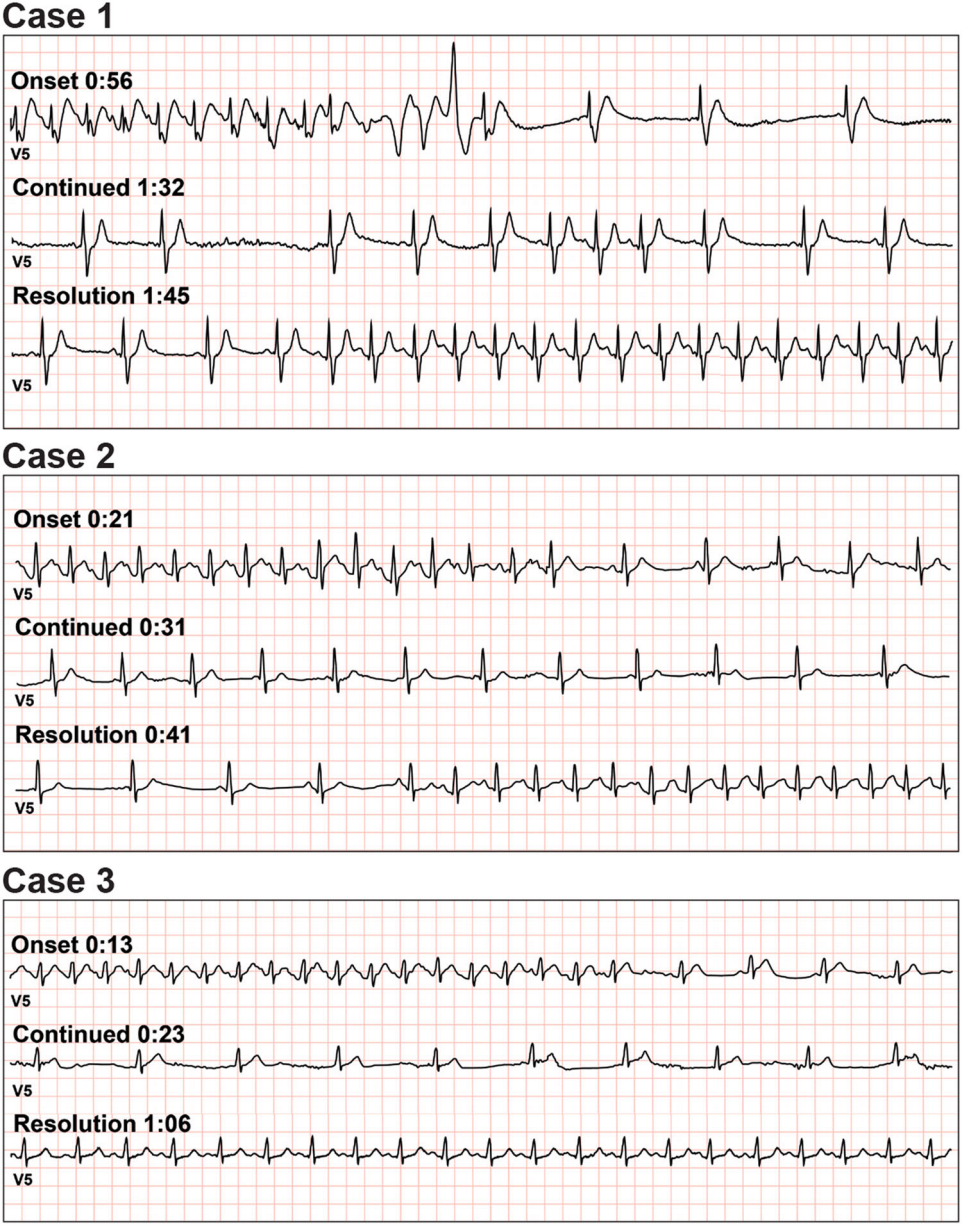
A series of single‐lead rhythm strips covering the syncopal event from onset to resolution are presented for each case. Times are postpeak exercise in active recovery at 1.7MPH/0% grade. Case 1. Electrocardiogram demonstrates a swift transition from sinus tachycardia to profound junctional bradycardia, likely due to intense vagal tone, occurring simultaneously with syncope. Note also a brief run of ventricular tachycardia thought to be an escape rhythm triggered by rapid heart rate slowing. Case 2. Electrocardiogram similarly demonstrating a sudden, vagally mediated decrease in heart rate in recovery with transient atrioventricular (AV) block occurring simultaneously with syncope. Case 3. Electrocardiogram demonstrating a sudden decrease in heart rate during recovery, occurring simultaneously with syncope. The abrupt slowing of the hazard ratio is likely vagally mediated.

We identified two additional cases of BJR syncope in women ages 32 and 29 with idiopathic Group 1 PAH occurring during CPET 15 and 26 months after the index case, respectively, with similar findings. Syncope occurred at 21 s postpeak exercise in active recovery in the second case and 13 s postpeak exercise as the patient stopped active recovery and attempted to sit down in the third case. Details of all three cases are described in Table 1, and the ECGs are presented in Figure 1. Treatment for all cases consisted only of minor adjustments to their medical programs. All are currently stable clinically, and two patients have performed repeat CPETs without syncope.

**Table 1:** Summary of general patient characteristics and exercise hemodynamic data

	Patient 1	Patient 2	Patient 3	Mean	SD
Age (years)	21	32	29	27.3	5.7
Body mass index—BMI (kg/m^2^)	28.3	25.7	44.3	32.8	10.1
Right ventricular systolic pressure (mmHg)	50	59	86	65.0	18.7
Peak V̇O_2_ (ml/kg/min)	19.2	21.1	11.7	17.3	5.0
Percent predicted V̇O_2_	51	63	34	49.3	14.6
V̇_E_V̇CO_2_ slope	36.3	42.0	43.3	40.5	3.7
Rest systolic blood pressure (mmHg)	90	110	146	115.3	28.4
Peak systolic blood pressure (mmHg)	142	142	154	146.0	6.9
Rest heart rate (bpm)	89	72	91	84.0	10.4
Peak heart rate (bpm)	193	184	173	183.3	10.0
Onset of syncope post‐peak exercise (s)	56	21	13	30	22.9
Heart rate at onset of syncope (bpm)	184	151	167	167.3	16.5
Minimum heart rate during syncope (bpm)	27	70	60	52.3	22.5
Change in heart rate (bpm)	157	81	107	115.0	38.6
Duration of loss of consciousness (seconds)	36	15	30	27.0	10.8
Time of full resolution (s)	52	25	48	41.7	14.6

During this time period of November 2018 through January 2021, a total of 26,162 exercise tests were performed in our laboratory, including 11,387 CPETs of which 373 were on patients with PAH. Thus, BJR syncopy was a relatively rare event, occurring in only 3/373 (0.8%) CPETs on patients with PAH during the 26‐month study period.

We propose a novel presentation of BJR syncope in the setting of exercise testing in PAH. We speculate that high right ventricular pressures and dilatation in pulmonary hypertension can cause interventricular septal shifting, resulting in impaired pulmonary venous return, decreased left ventricular filling, and inadequate cardiac output.^7^, ^8^, ^9^ Coupled with exercise, the underfilled but hyperdynamic left ventricle has a markedly reduced end‐systolic volume essentially leading to near‐total left ventricular collapse. This phenomenon is further accentuated by decreased venous return with lower extremity pooling of blood following the cessation of vigorous exercise.^10^ We hypothesize that unique hemodynamic circumstances of pulmonary hypertension can result in mechanical stimulation of cardiac receptors, which ultimately activate afferent vagal fibers. The resultant sudden vagal storm leads to bradycardia, hypotension, and ultimately syncope.

Our three patients exhibited an almost instantaneous fall in HR of 115 ± 39 bpm followed by loss of consciousness suggesting an intense vagal storm. All reported symptoms consistent with underfilling of the left ventricle before the abrupt drop in HR. Full recovery of consciousness—and HR—without neurologic sequelae ensued within 1 min (42 ± 15 s).

We believe that these syncopal events were not likely mediated through aortic baroreceptors. HR was still high before syncopy, suggesting high sympathetic tone. The drop in HR was immediate and profound.

Syncope occurring during exercise raises concern over ventricular arrhythmias and spontaneous AV block. None of the subjects had bradyarrhythmias or tachyarrhythmias of concern during exercise. One subject did exhibit a brief (3‐beat) run of NSVT at the onset of syncope which we interpret as an escape rhythm in response to a sudden profound drop in HR rather than a cause of the syncope. We frequently see 3–5 beat runs of NSVT on exercise tests (3.1% of all tests performed) but have never observed syncope or noted patient reports of presyncope with an isolated brief run of VT. Certainly, NSVT can result in retrograde AV conduction and subsequently disrupt anterograde AV conduction of sinus impulses. However, the duration of bradycardia in this example well exceeds the duration of NSVT, so we favor the BJR mechanism proposed.

We have previously reported on spontaneous second‐degree AV block during exercise (19 cases in 40,715 exercise tests = 0.05%),^11^ and AV block at an intra‐ or infrahisian level—if profound—could potentially precipitate syncope. In our current cases, however, we saw no persistent AV block during the periods of bradycardia—rhythm was mostly 1:1 AV conduction or junctional—after the sudden drop in HR. None of the patients had arrhythmias on their resting ECGs or any known history of AV block. Thus, our clinical suspicion for intrahisian or infrahisian disease as a cause of syncope in our cases was low. It is important to emphasize that syncope occurred simultaneously with, not after, slowing of HR.

CPET is indicated for risk assessment and to inform therapeutic decisions and evaluate response to therapy in PAH.^12^, ^13^ In both adults and children diagnosed with PAH, CPET has been proven safe with minimal associated risks.^14^, ^15^ When syncope does occur during heavy exercise, however, it places the patient in an intermediate risk category.^13^ As described above, these syncopies were relatively rare events, occurring in only 0.8% of CPETs on patients with PAH.

In conclusion, although classically described following the administration of intravenous veratrum alkaloids and inferior myocardial infarction, we here present a novel description of BJR in the context of CPETs in patients with Group 1 PAH. Three BJR syncopies in young women with Group 1 PAH were identified, resulting in a rate of 0.8% of tests on patients with PAH evaluated during the time frame covering these events. Laboratories conducting treadmill exercise tests on patients with PAH should be aware of the possibility of BJR syncope during active recovery and consider evasive action—terminating further exercise with immediate transfer to supine recovery to enhance venous return and reduce sympathetic tone—to prevent syncope and possible injury when PAH patients complain of dizziness or other signs of inadequate left ventricular filling despite high HR.

## AUTHOR CONTRIBUTIONS

**Kolade M. Agboola**: Investigation, writing–original draft, writing–review and editing, visualization. **Gurukripa N. Kowlgi**: Investigation, writing–original draft, writing–review and editing. **Ibolya Csecs**: Writing–original draft, writing–review and editing. **Hilary M. DuBrock**: resources, writing–original draft. **Hector R. Cajigas**: Resources, writing–original draft. **Thomas G. Allison**: Conceptualization, methodology, validation, formal analysis, resources, data curation, writing–original draft, writing–review and editing, visualization, supervision, project administration.

## CONFLICT OF INTEREST

The authors declare no conflict of interest.

## ETHICS STATEMENT

Query of the exercise testing database was conducted under the Mayo Clinic Institutional Review Board approved study number # 17‐004494.
